# Mitigate lumbar crash injury of reclined occupants with assisted repositioning device

**DOI:** 10.3389/fbioe.2025.1540569

**Published:** 2025-03-27

**Authors:** Wenxuan Shen, Qing Zhou

**Affiliations:** ^1^ School of Vehicle and Mobility, Tsinghua University, Beijing, China; ^2^ State Key Laboratory of Intelligent Green Vehicle and Mobility, Tsinghua University, Beijing, China

**Keywords:** reclined occupant, occupant repositioning, lumbar injury prevention, human body model, occupant restraint system

## Abstract

In autonomous vehicles, reclined seating positions are increasingly popular for improving occupant comfort, but they also pose significant challenges for crash safety, especially concerning lumbar injury risks. This study investigated the potential of assisted repositioning to mitigate lumbar injuries in reclined occupants during frontal collisions. Assisted repositioning combined active intervention with collision inertia to return reclined occupants to an upright posture prior to peak lumbar loads. THUMS simulations at crash severities from 30 km/h to 70 km/h demonstrated that assisted repositioning could significantly reduce lumbar injury risk, particularly at 50 km/h, where the risk decreased from 66.9% to 32.4%. To provide whole-body protection, this study introduced two solutions for coordinating repositioning devices with the conventional three-point seatbelt. The “active solution” utilized an active lumbar support for repositioning, while the “passive solution” employed a lumbar airbag. Both solutions emphasized the need for careful coordination of occupant repositioning with seatbelt functions to optimize safety across different body regions. These findings presented a new strategy for enhancing crash protection in flexible cabin configurations, contributing to occupant safety in future autonomous vehicles.

## 1 Introduction

Autonomous driving may allow more flexible vehicle cabin layout and comfortable riding experience. For example, on self-driving robotaxi, occupants may prefer a reclined seating position. In fact, “zero-gravity” seats have now been available in some vehicles to enhance comfort. However, existing occupant restraint systems, designed for upright occupants, are not effective in protecting reclined occupants. According to NASS-CDS data, the fatality rate for occupants in a fully reclined position in frontal crashes is 77% higher compared to those in upright position (Dissanaike et al., 2008). Considering that autonomous driving vehicles cannot completely avoid collisions (Mueller et al., 2020; Pilet et al., 2021; Sun et al., 2023), crash protection of reclined occupant is becoming a new challenge in automotive crash safety.

In frontal collision accidents, since reclined occupants are prone to submarining and the crash load has a greater component along the axial direction of spine, main safety concerns are spinal injuries and submarining. Studies in recent years have shown that the reclined posture places the lumbar spine at a higher risk of injury. Uriot et al. (2015) observed lumbar and iliac wing fractures in post-mortem human subject (PMHS) tests conducted for the occupant submarining study. The University of Virginia (UVA) conducted a frontal impact PMHS test for a 50 km/h frontal impact at a 50° angle of recline (Richardson et al., 2020a; Richardson et al., 2020b; Richardson et al., 2020c). It was found that the lumbar spine and pelvis were subjected to greater loads than in an upright position, increasing the risk of lumbar vertebra and iliac wing fractures. The University of Michigan Transportation Research Institute (UMTRI) conducted PMHS tests (from the National Highway Traffic Safety Administration Biomechanical Testing Database) at 32 km/h at two recline angles of 25° and 45°, and the results showed that the risk of thoracolumbar spine fracture increased with higher recline angles. Baudrit et al. (2022) performed PMHS tests on subjects with a recline angle of 58° at a crash severity of 13.4 m/s and compared the results to the upright seated PMHS tests performed by Uriot et al. (2015). The results showed a significant number of lumbar and pelvic injuries. All these results indicate that reclined occupants are at a high risk for lumbar and pelvic injuries. On the other hand, there also have been in-depth researches regarding submarining injuries (Boyle et al., 2019; Gepner et al., 2019; Rawska et al., 2019). Reclined occupants have a greater initial angle of the anterior superior iliac spine (ASIS), making the ASIS more likely to slide out of the lap-belt during collisions, and therefore are at greater risk of submarining.

Currently, there are no developed solutions for mitigating lumbar injuries of reclined occupants under collisions. One of the main proposed strategies is active repositioning. Baumann et al. (2001) suggested that collision injury risk could be reduced by adjusting the seat from a reclined to a normal seating position once an unavoidable collision is detected. Östh et al. (2020) evaluated this strategy using human body model (HBM) simulations, in which the reclined seat back was actively rotated back to adjust the occupant’s posture before a collision. However, due to the flexibility of the lumbar spine, the pelvis could not fully return to the upright angle, making it difficult to prevent submarining and potentially exacerbating lumbar spine flexion deformation. Ji et al. (2017) were the first to propose using the inertia of the collision to adjust the posture of reclined occupants. By moving the knee bolster near the occupant’s knee before the collision, the hip movement is effectively limited, leading the upper body to an upright sitting position by the impact inertia. This method addresses both the submarining and lumbar injury of reclined occupants at a collision severity of 30 km/h. Additionally, Östling et al. (2022) utilized the seat track load limiter strategy with energy dissipation and verified the protective effect on occupant lumbar injury through both tests (Östling et al., 2021) and simulations using the THOR dummy.

Previous studies have highlighted the increased risk of lumbar injury in reclined occupants and the lack of recognized protective countermeasures. This study focused on lumbar spine protection for reclined occupants. By analyzing the kinematic and kinetic responses of the reclined occupant’s spine, the protection mechanisms of occupant repositioning were demonstrated in detail. After evaluating the advantages and disadvantages of existing protection methods, we proposed a new occupant repositioning scheme, termed “assisted repositioning”, which combines an active device with the inertial effect during a collision. The scheme was verified through THUMS simulations. Whole-body solutions were developed for a reclined occupant at a crash severity of 50 km/h, integrating the assisted repositioning scheme with the three-point seatbelt system.

## 2 Methods and concepts

### 2.1 THUMS evaluation

Human body model simulation was the main research tool in this study. The THUMS human body model used for crash simulation has been validated against post-mortem human subject (PMHS) tests. The reclined THUMS model was generated using a repositioning method developed by our group to match the specific spinal alignment of the PMHS subject (Shen et al., 2023; Tang et al., 2023). The PMHS subjects were subjected to a 50 km/h crash pulse and involved a restraint system designed to reduce the risk of submarining in reclined seating (Östling et al., 2017). In simulation of the PHMS test, the reclined THUMS model could well capture the kinematic trend of the subject in the XZ plane. In terms of injury response, the model predicted a lumbar vertebra to have the highest risk of injury, and that was the vertebra actually fractured in the test.

### 2.2 Lumbar injury mechanism and injury risk function

Based on the simulation results of the validated THUMS model, lumbar injury mechanism can be analyzed through the kinematic response process of the thoracolumbar spine. When reclined occupant is under frontal impact load, initially, the thoracolumbar spine undergoes compression along the axial direction of the spinal structure. Then, due to the overall forward collision inertia, the spinal compression stage reaches its end, and the spinal flexion gradually dominates the kinematics.

According to the lumbar injury theory of Tushak et al. (2022), the lumbar injuries result from the combined compression and flexion of the spine column, which posited that the injury risk of a lumbar vertebra is primarily determined by the compression force and flexion moment. The compression forces of all lumbar vertebrae peak at the end of the spinal compression stage (the first stage of the structural response of the spine). Due to the subsequent process of flexion deformation (the second stage), the flexion moments of the lumbar vertebrae from L1 to L5 peak successively in the spinal flexion stage. Consequently, L1 experiences the most intense load due to the closest time interval between peak force and peak moment.

The injury risks of L1-L5 were further assessed using the lumbar injury risk function developed by Tushak et al. (2022). The injury risk function, expressed as a predictor variable, is based on a linear combination of axial compressive stress (force divided by cross-sectional area: CSA) and flexion moment (moment divided by CSA^3/2^), denoted as L (Equation 1). The function was used to evaluate the risk of injury for each vertebra. Additionally, the function incorporates a weighting factor, α, to consider the relative contribution to damage between force and moment. Furthermore, age (in years) is included as a covariate, with the coefficient values, $\beta_{0}\text{ and }\beta_{1}$ , provided in Equation 2.

$$L_{fx}\left(F,M\right)=\left(1-\alpha \right)\frac{F}{CSA}+\alpha \frac{M}{{CSA}^{3/2}}$$ (1)

$$P\left(fracture|L_{fx},Age\right)=1-e^{{-\left[\frac{L_{fx}}{e^{\beta_{0}+\beta_{1}Age}}\right]}^{\lambda }}$$ (2)

α=0.11

$$\beta_{0}=1.89043$$

$$\beta_{1}=-0.00086$$

λ=1/0.201

### 2.3 Concept of assisted occupant repositioning

The lumbar injury mechanism has spurred a design idea of protective measures for reclined occupant. Controlling the kinematic response of the spine is key to reduce the risk of lumbar injury. Analyzing from a mechanical point of view, the spinal vertical component of the inertial force leads to the inertial return, while the axial component causes spinal compression and rapid accumulation of lumbar injury risk. Therefore, we need to find a way to “enhance” the vertical component of the inertial force to steer the spine out of axial compression and into a relatively safe mode in which the spine rotates around the pelvis. As the spine transitions into fixed-axis rotation, the axial direction of the spine changes, and because the direction of the inertial force is constant, its vertical component increases and its axial component decreases, further guiding the spine into fixed-axis rotation.

Our method is to implement active devices for assistance in the initial stage of the collision on the “inertial repositioning”, providing force in the spinal vertical direction to promote the reclined occupant’s spine out of the axial compression mode as soon as possible, called “assisted repositioning”. Traditional restraint systems can then be used for collision protection once the occupant has already returned to an upright sitting posture. In this study, a special action point is to determine so that when the vertical spinal force is applied at this location, the effect of controlling the overall spinal kinematic response can be achieved. Based on the observation and analysis of several crash simulation cases of reclined occupants, it is assumed that the specific action point of the assisted repositioning device should be the inflection point of the S-curve of the initial spinal alignment, which is often located in the lumbar spine of the occupant, and the inflection point is located near the L1 vertebra in the model used in this study.

### 2.4 Sled test environment and restraint system

**FIGURE 1:**
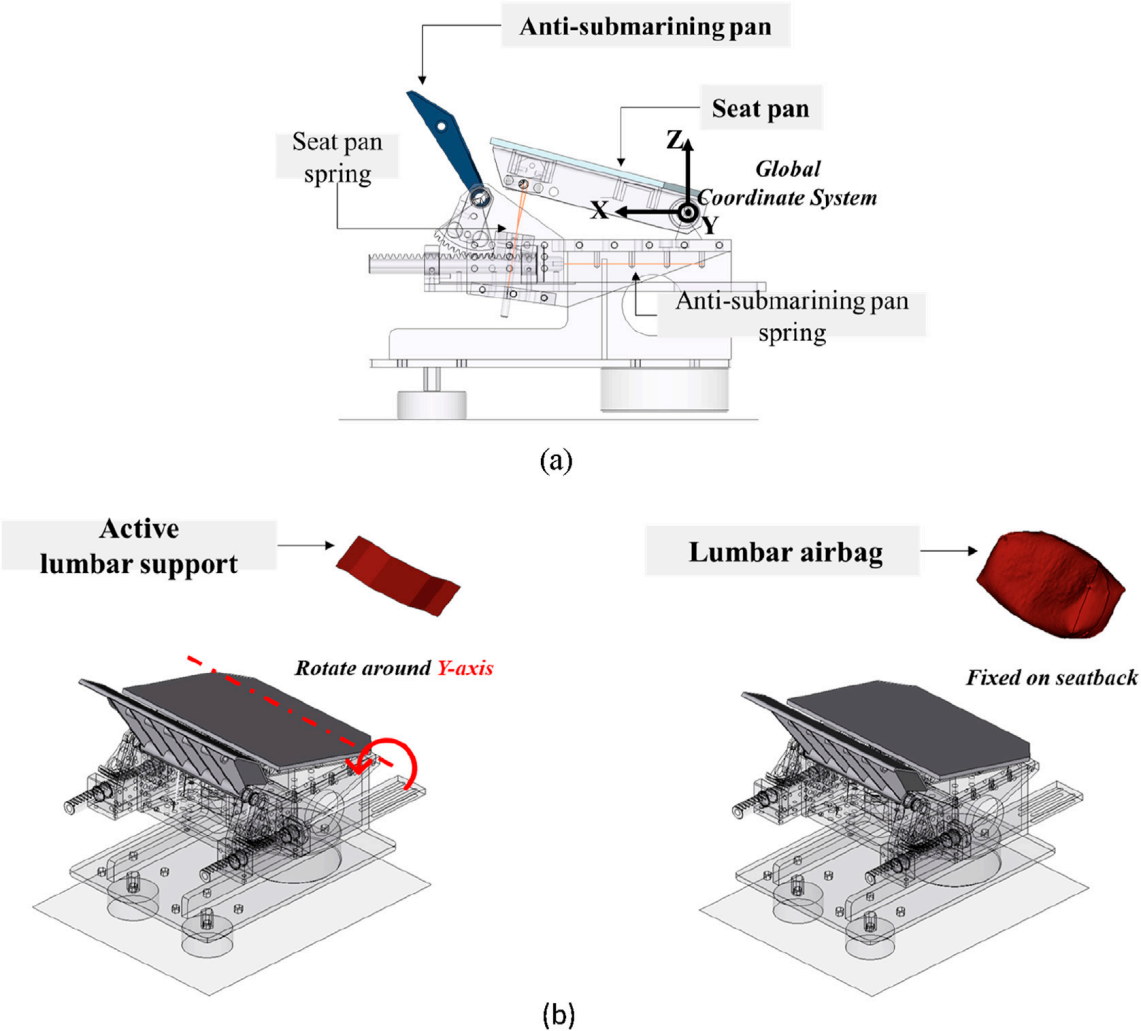
Restraint system and global coordinate system. **(A)** Semi-rigid seat and global coordinate system. **(B)** Assisted repositioning devices.

**FIGURE 2:**
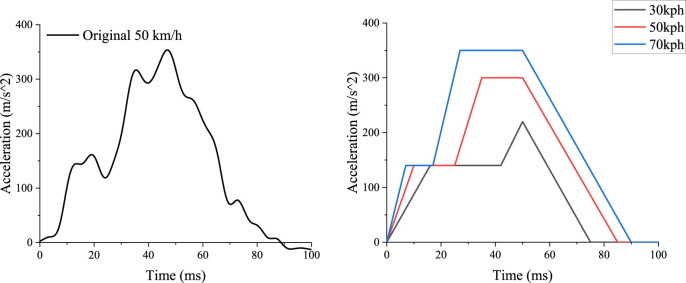
Full frontal rigid barrier pulse with initial velocity of 50 km/h (Richardson et al., 2020) and the 30 km/h and 70 km/h crash pulses scaled from the simplified 50 km/h pulse.

The restraint system in simulation matched the setup used in the PMHS test (Richardson et al., 2020a), which featured a semi-rigid seat and an anti-submarining three-point seatbelt system. The semi-rigid seat model (Figure 1) has been widely used in related studies. It was mainly comprised of a rigid seat pan and an anti-submarining pan with adjustable spring stiffness. The spring stiffness was adjusted and validated to ensure the seat conformed to the response of the front seat in a vehicle (Uriot et al., 2015). The three-point seatbelt system was outfitted with dual lap-belt pretensioners, a pretensioner at the shoulder-belt retractor, a locking tongue at the seatbelt buckle, and a shoulder-belt load limiter of 3.5 kN. As part of a seat-mounted seatbelt system, the D-ring was positioned at the seatback. The seatbelt system was intended to reduce the risk of submarining in reclined seating (Östling et al., 2017) and was set up in this study according to Richardson et al. (2020a) using the Seatbelts module in Oasys PRIMER. A 30 g acceleration pulse from the PMHS test was used in the simulation environment (Figure 2). Its initial impact velocity was 50 km/h.

The global coordinate system was defined in Figure 1. The origin was the midpoint of the seat pan axis. The longitudinal X-axis was directed forward, the lateral Y-axis was directed to the subject’s left, and the vertical Z-axis was directed upward.

The active device for assisted repositioning was modeled as a rigid plate or an airbag in the simulation environment, referred to as the active lumbar support or the lumbar airbag, as shown in Figure 1. The lumbar support was a rigid plate measured 67 mm in height and 248 mm in width, positioned near the L1 vertebra of the THUMS model initially, and was constrained to rotate about a fixed axis around the seat pan axis using the *CONSTRAINED_JOINT_REVOLUTE keyword. The lumbar airbag was fixed in a similar position and measured 100 mm high, 248 mm wide, and 100 mm in length when fully inflated. In the physical layout, the active device could be integrated into the seatback, with active motion of the lumbar support independent of seatback rotation.

During the repositioning process driven by impact inertia, the time for occupant torso’s movement from a reclined posture to an upright seating position was about 65 ms. With the assistance of the repositioning device, it was expected that the occupant should complete the repositioning process in less than 50 ms. Upon these analyses, a 200 Nm Y-moment was applied to the lumbar support from 0 to 50 ms. For the lumbar airbag, there were two settings for ignition time: 0 ms and 20 ms. The 0 ms start time for both devices indicated that this represented an active safety solution. The 20 ms ignition time represented a scenario in which the airbag may be commanded by the same ECU that controls the frontal collision airbag, making this setting representative of a passive solution. To facilitate the preliminary study, the lumbar support was employed to evaluate the effectiveness of assisted repositioning in mitigating the risk of lumbar injury for reclined occupants.

### 2.5 Crash pulse scale

To investigate the performance of the protection measure under different collision severities, crash pulses with initial velocities of 30 km/h and 70 km/h were derived from that of 50 km/h using the double trapezoidal pulse simplification and a scaling method (Figure 2). The scaling method (Woolley, 2008) operated by scaling the crash energy according to initial velocity and obtaining the corresponding maximum displacement through the kinetic energy-displacement relationship. By maintaining the same acceleration-displacement relation in the loading stage of the collision, the crash pulses for the loading stage were computed for different initial velocities. The pulses for the unloading stage were obtained by intercepting and translating the pulses from the 50 km/h pulse. This method ensured that the energy dissipation is proportional to the initial kinetic energy, and the stiffness during the loading stage remained the same (assuming with respect to the same vehicle structure).

### 2.6 Simulation matrix for evaluation of assisted repositioning

The calibrated HBM model with conventional three-point seatbelt restraint setup was selected as the reference case, termed “normal restraint”. To validate the inertial return theory, which suggests that removing the shoulder-belt can reduce lumbar load for reclined occupants, the “inertial repositioning” condition was set up with the lap-belt as the only restraint. This condition was essential for the subsequent application of the assisted repositioning concept. For the “assisted repositioning” condition, the restraint system combined the lap-belt with the active lumbar support. The active lumbar support was added to the normal restraint setup, creating the “coupled restraint” condition. This condition was established to examine the interaction between the assisted repositioning device and the conventional restraint system.

**FIGURE 3:**
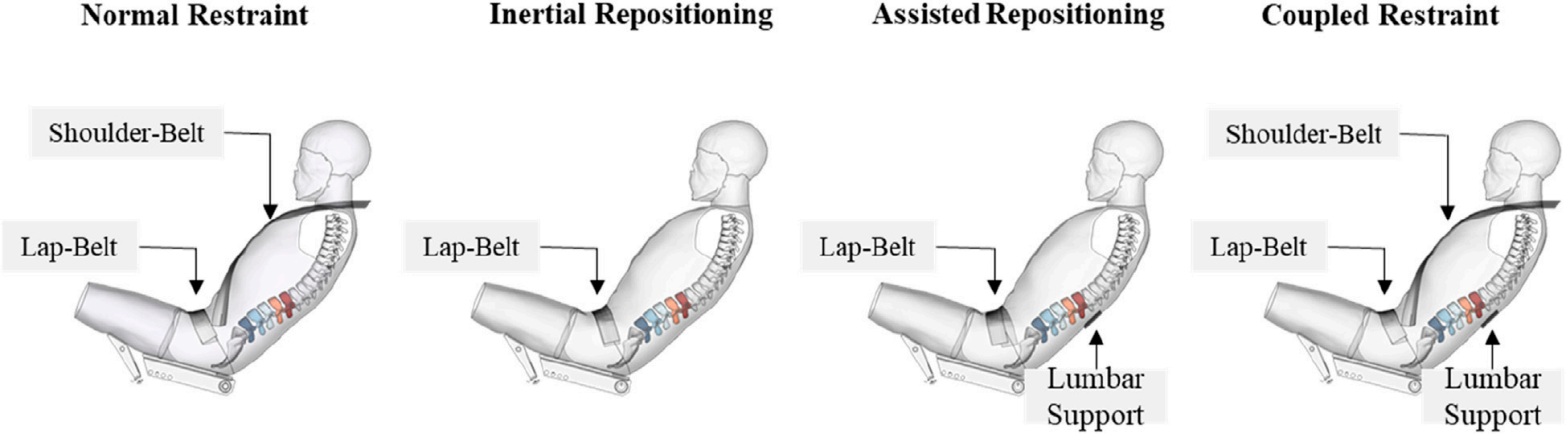
Four restraint setups for each group.

In total, there were four conditions in the 50 km/h simulation group, as shown in Figure 3. Using the scaled crash pulses for 30 km/h and 70 km/h, two additional simulation groups were created, resulting in a total of 12 simulation cases. The purpose of this 3-velocity group design was to investigate the sensitivity of lumbar injury of reclined occupant to impact severity and to assess the effect of impact severity on the protective efficacy. Additionally, it allowed a quantitative analysis of the shoulder-belt’s impact on lumbar injury of reclined occupant.

As the simulation of normal restraint in 50 km/h has been validated, this study mainly focused on the 50 km/h scenario.

### 2.7 Simulation setups for whole-body solutions

**FIGURE 4:**
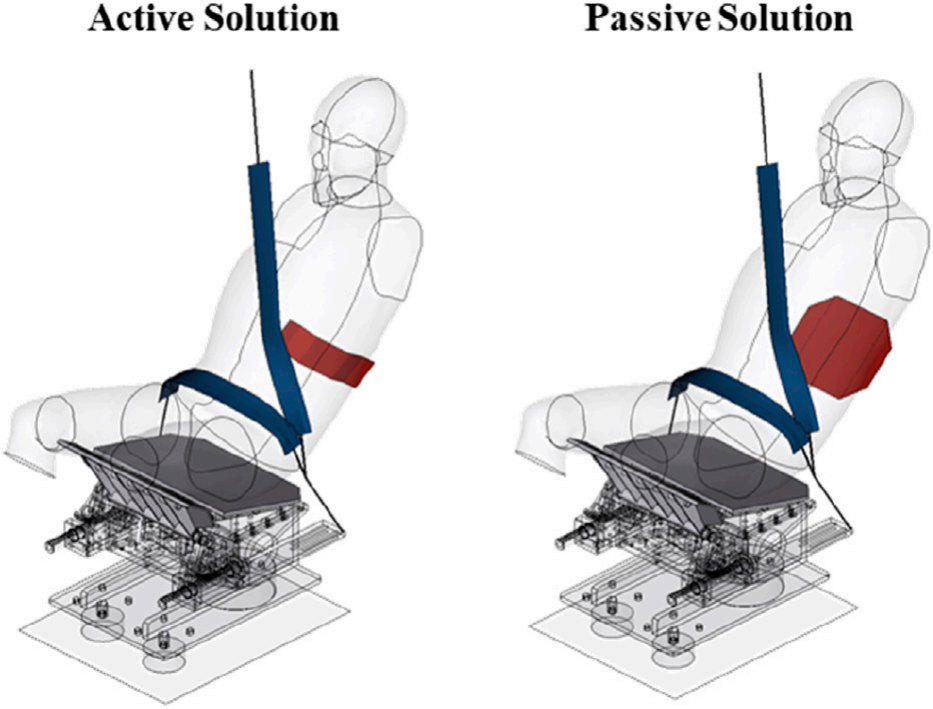
Restraint system for whole-body solutions.

This study presented two whole-body solutions under 50 km/h to demonstrate the application potential of the assisted repositioning device: an active solution and a passive solution. The active solution utilized the lumbar support, while the passive solution employs a lumbar airbag inflated at 20 m. For coordination between the three-point seatbelt system and the assisted repositioning device, seatbelt settings were modified based on the PMHS test. The D-ring was repositioned to approximate the position of a B-pillar integrated D-ring, which prevented contact between the shoulder-belt and the reclined occupant’s upper body during the repositioning process. For the lap-belt, the limiting force for the lap-belt retractor was reduced to 8 kN to enhance iliac protection. The semi-rigid seat model and the THUMS model remained unchanged. An overview is shown in Figure 4.

In order to assess the whole-body injury of the occupants, HIC (Head Injury Criteria) was output for the head injury, BrIC (Brain Injury Criteria) and CSDM (Cumulative Strain Damage Measure with a 0.2 strain threshold) were output for the brain, and Nij was output for the neck. The rib fracture risk was calculated using a strain-based probabilistic method based on peak strains in the cortical bone of each rib (Forman et al., 2022) and the Weibull-smoothed injury risk function. Cross-sectional outputs of the left and right iliac crests were defined as well. The resultant force was considered as the injury predictor for iliac fracture, which was a typical injury type observed in PMHS tests.

## 3 Results

### 3.1 Simulation results in evaluation matrix

**FIGURE 5:**
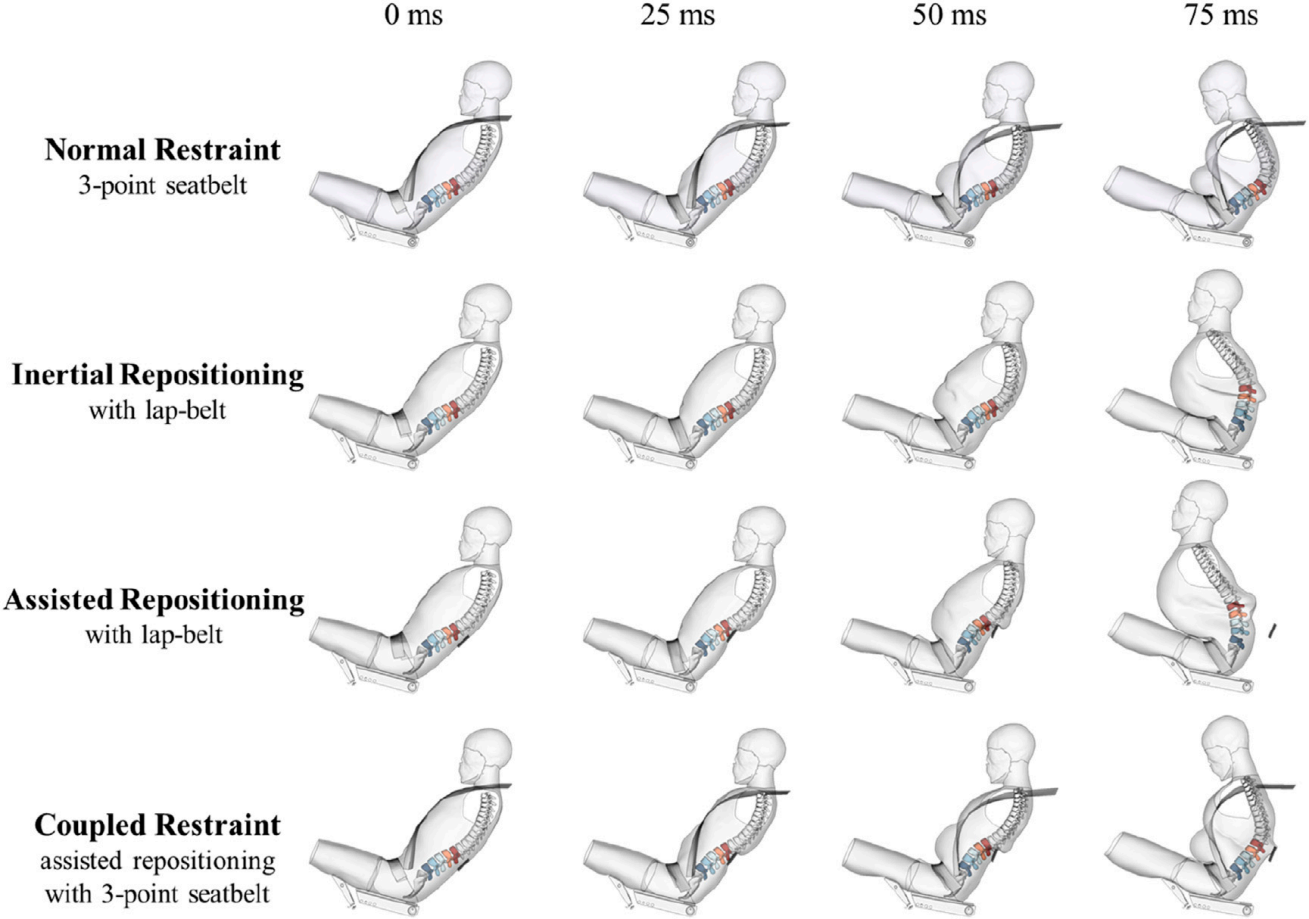
Kinematic responses under impact of 50 km/h.

Figure 5 shows the overall and the thoracolumbar spinal kinematic responses of the reclined occupant for each case in the 50 km/h group in the ZX plane.

**FIGURE 6:**
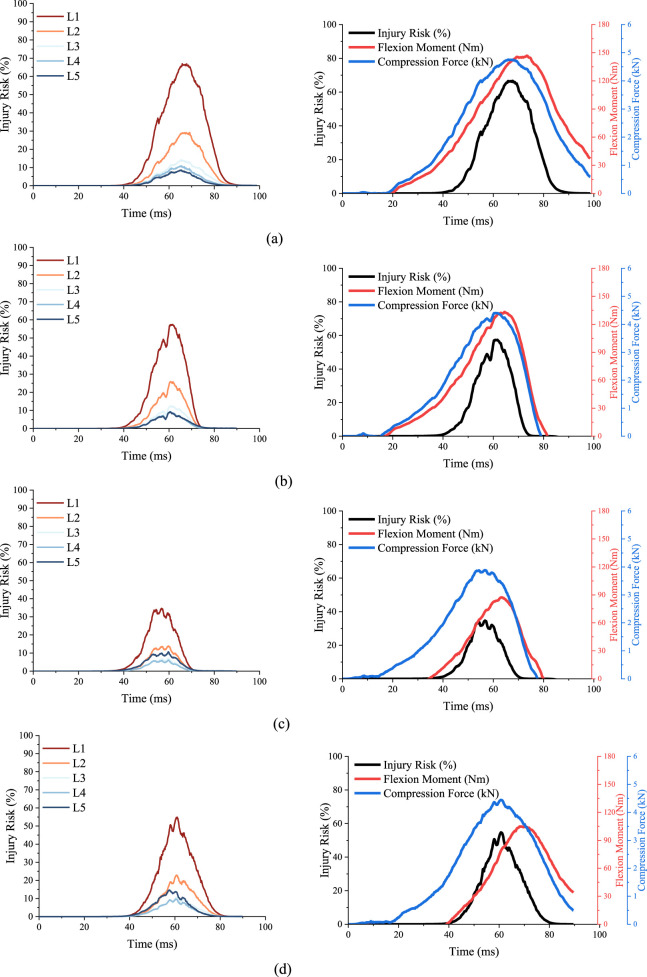
Time histories of lumbar injury outputs of cases in the 50 km/h group. **(A)** Injury risks of lumbar vertebrae and mechanical responses of L1 in normal restraint case. **(B)** Injury risks of lumbar vertebrae and mechanical responses of L1 in inertial repositioning case. **(C)** Injury risks of lumbar vertebrae and mechanical responses of L1 in assisted repositioning case. **(D)** Injury risks of lumbar vertebrae and mechanical responses of L1 in coupled restraint case.

For reclined occupants under 50 km/h frontal impact, time histories of lumbar injury outputs under the four restraint conditions, including compression forces, flexion moments and injury risks, are displayed in Figure 6. As shown in the lumbar injury risk curves, the L1 vertebra suffers the highest injury risk among all conditions, so only the compression forces and flexion moments of L1 are plotted. In the normal restraint case, the peak of compression force and flexion moment are 4.8 kN and 148 Nm, respectively. According to the injury risk function mentioned above, the highest lumbar fracture risk is 66.9%. In the case of the inertial repositioning, the peak of compression force and flexion moment are 4.4 kN and 133 Nm, respectively, corresponding to a 54.3% lumbar injury risk. In the case of the assisted repositioning, the peak of compression force and flexion moment are 3.9 kN and 87 Nm, respectively, corresponding to a 32.4% lumbar injury risk. In the case of the coupled restraint, the peak of compression force and flexion moment are 4.4 kN and 105 Nm, respectively, corresponding to a 51.8% lumbar injury risk.

**FIGURE 7:**
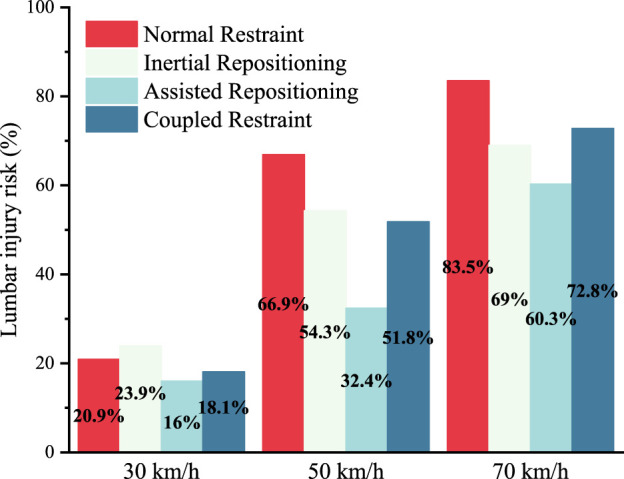
Lumbar injury risks of all 12 cases.

In the 30 km/h and 70 km/h crash simulation groups, L1 still has the highest lumbar injury risk. Table 1 shows the peak values of compression forces, flexion moments and injury risks of L1 in those eight cases. As shown in Figure 7, in all three groups, the assisted repositioning cases exhibits the lowest risks of lumbar injury.

**TABLE 1:** Simulation outputs of each case in the 30 km/h and 70 km/h groups.

Case	30 km/h				70 km/h			
	Normal	Inertial	Assisted	Coupled	Normal	Inertial	Assisted	Coupled
Compression Force (kN)	3.4	3.5	3.2	3.4	5.2	4.7	4.5	4.9
Flexion Moment (Nm)	106	109	78	87	155	137	99	123
Injury Risk	20.9%	23.9%	16.0%	18.1%	83.5%	69.0%	60.9%	72.8%

### 3.2 Effect of inertial repositioning

A comparison in the simulation results between the normal restraint and the inertial repositioning has demonstrated the protective effect of the “inertial return” concept. The time histories reveal that under the normal restraint condition, the peak lumbar injury risk reaches 66.9%, accompanied by a peak compression force of 4.8 kN and a peak flexion moment of 148 Nm. The peak lumbar injury risk of L1 under the inertial repositioning is 54.3%, which represents a 12-point (percentage point) reduction compared to the normal restraint condition. The peak compression force and flexion moment are reduced by 8.3% and 10.1%, respectively.

The effect of inertial repositioning was further examined across different crash severities. At a speed of 70 km/h, the inertial repositioning mechanism reduced the risk of lumbar spine injury for the reclined occupant by 15 points., from 83.5% to 65.0%. Notably, a lumbar injury risk of 65.0% under inertial repositioning at 70 km/h is comparable to the risk associated with normal restraint at a crash severity of 50 km/h. However, at a lower crash severity of 30 km/h, the inertial repositioning does not demonstrate any benefit for the lumbar spine. Under normal restraint conditions, the lumbar spine injury risk is 20.9%, whereas under inertial repositioning conditions, it increases to 23.9%. Our explanation for this observation is that at a collision speed of 30 km/h, the kinetic energy is significantly lower, and a substantial portion is dissipated by the retractor’s energy absorption and deformation of the occupant’s soft tissues, particularly with the shoulder-belt in place.

### 3.3 Effect of assisted repositioning

Building on an in-depth understanding of the mechanisms of lumbar injury of reclined occupants and the protective effects of inertial repositioning, this study introduced the concept of assisted repositioning. This approach involved using active lumbar support to intervene in the spinal crash response, aiding the collision inertia to expedite the repositioning of the occupant’s upper body to upright position. This method was aimed to achieve an enhanced lumbar injury mitigation under frontal collision.

For crash severity of 50 km/h, as shown in Figure 5, the kinematic response at 50 m shows significantly lower axial deformation of occupant’s spine under the assisted repositioning condition compared to that under the normal restraint condition. The repositioning of the upper body is substantially greater, and the posture control of spinal collision response is better achieved relative to inertial repositioning. With the assisted repositioning, the lumbar injury risk for the reclined occupant assisted repositioning is 35 points lower than under the normal restraint condition and 22 points lower than under the inertial repositioning condition, indicating a much more effective protective effect.

For crash severities of 30 km/h and 70 km/h, assisted repositioning also demonstrates a significant reduction in lumbar injury risk. At 30 km/h, the lumbar injury risk for the reclined occupant under assisted repositioning is reduced by five points compared to the normal restraint condition and by eight points compared to the inertial repositioning condition. Under the 70 km/h crash severity, the risk of lumbar spine injury for reclined occupants is reduced by 23 points compared to the normal restraint condition and by nine points compared to the inertial repositioning condition.

Comparing the lumbar injury risk across the three simulation groups at different crash severities, it reveals that at 30 km/h, the risk remains low (25% or less) for reclined occupants, irrespective of lumbar protection. At 70 km/h, although assisted repositioning reduces the lumbar injury risk by at least 23 points, it is still difficult to lower the risk below 60%. Conversely, at 50 km/h, assisted repositioning significantly reduces the lumbar injury risk from 66.9% to 32.4%, transitioning from a high to a low risk level. This suggests that the effect of assisted repositioning is closely related to crash severity. At 30 km/h, where the lumbar injury risk is already low, its impact is not significant, and at 70 km/h, where the risk is too high, it is insufficient. A crash speed around 50 km/h appears to be the optimal scenario for the application of assisted repositioning.

### 3.4 Effect of shoulder-belt

The simulations conducted in this study have clearly demonstrated the negative effect of the shoulder-belt on reclined occupants in a quantitative way. Specifically, among the four simulated restraint conditions, the normal restraint case and the inertia repositioning case are grouped for analysis, while the coupled restraint case and the assisted repositioning case are grouped into another set. Thus, the difference in lumbar injury risk within each group is influenced solely by the shoulder-belt restraint. Table 2 shows the negative impact of shoulder-belt on lumbar injury risk at different crash severities. The average increase in lumbar injury risk due to the shoulder-belt is −0.9% (30 km/h), 16.0% (50 km/h) and 13.5% (70 km/h).

**TABLE 2:** Effect of shoulder-belt on lumbar injury risks of the reclined occupant.

Case		Shoulder-belt	Lumbar support	Lumbar injury risk	Effect of shoulder-belt
30 km/h	Normal Restraint	√	×	20.9%	−3.9%
	Inertial Repositioning	×	×	23.9%	
	Assisted Repositioning	×	√	16.0%	+2.1%
	Coupled Restraint	√	√	18.1%	
50 km/h	Normal Restraint	√	×	66.9%	+12.6%
	Inertial Repositioning	×	×	54.3%	
	Assisted Repositioning	×	√	32.4%	+19.4%
	Coupled Restraint	√	√	51.8%	
70 km/h	Normal Restraint	√	×	83.5%	+14.5%
	Inertial Repositioning	×	×	69.0%	
	Assisted repositioning	×	√	60.3%	+12.5%
	Coupled Restraint	√	√	72.8%	

### 3.5 Results of whole-body solutions

An active and a passive solution are presented to formulate an overall crash protection strategy for reclined occupants under a 50 km/h frontal crash. The injury profiles of the reclined occupant for each solution are summarized in Table 3.

**TABLE 3:** The injury profiles of the reclined occupant for each solution.

Case	Lumbar injury (%)	Iliac force (kN)	Head HIC	Brain BrIC	Brain CSDM	Neck N_ij_	Thorax AIS 3+ (%)
Normal Restraint	66.9	5.1	292	0.64	0.01	0.49	24.9
Active Solution	29.7	4.3	434	0.96	0.13	0.53	30.2
Passive Solution	42.0	4.3	190	0.40	0.01	0.33	29.5

**FIGURE 8:**
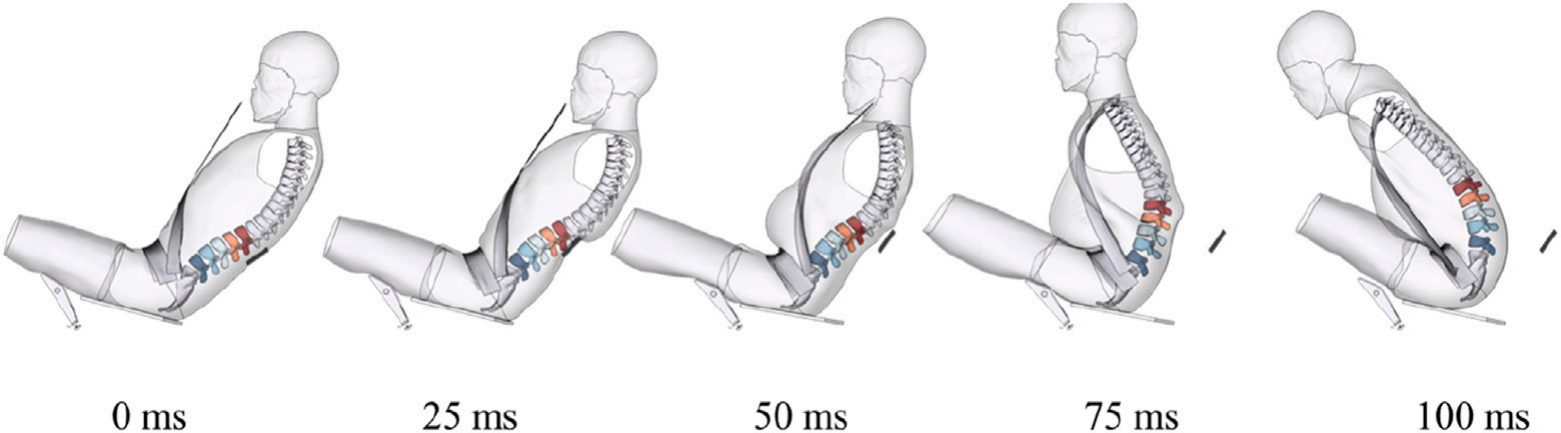
Kinematic response of reclined occupant under active solution.

In the active solution, simulation results show that the lumbar injury risk under this condition is 29.7%, reduced by 37 points from that of the normal restraint condition, and the peak iliac force was reduced by 16.1%, from 5.1 kN to 4.3 kN, effectively optimizing the occupant’s overall spinal injury risk to an acceptable level. Other injury parameters included an HIC of 434, 48.6% higher than that in normal condition but still within the regulatory threshold of 500, a BrIC of 0.96 and a CSDM of 0.13, indicating a notably 40% higher probability of AIS 3+ brain injury risk (Takhounts et al., 2013). The kinematic response indicated that the shoulder-belt restraint caused relative rotation between the head and shoulder, leading to high BrIC and CSDM. The thorax injury AIS 3+ probability is 30.2%, slightly higher than 24.9% in the normal condition, due to the shoulder-belt contacting the chest at the middle stage of the collision with significant relative velocity. Figure 8 shows the kinematic response of reclined occupant under the active solution.

**FIGURE 9:**
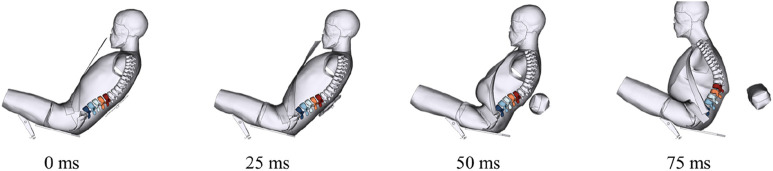
Kinematic response of reclined occupant under passive solution.

In the passive solution, simulation results indicate a lumbar injury risk of 42.0%, which remained 25 points lower than that in the normal restraint condition. While an increase in lumbar injury risk compared to the active solution was expected, significant improvement was observed in the metrics for head and neck injuries even compared to the normal restraint condition, including reductions of 34.9% in HIC, 37.5% in BrIC, and 32.7% in Nij. This improvement may be attributed to the slower repositioning and the more homogeneous load, which resulted in reduced relative motion between the head and chest. Figure 9 shows the kinematic response of reclined occupant under the passive solution.

## 4 Discussion

**FIGURE 10:**
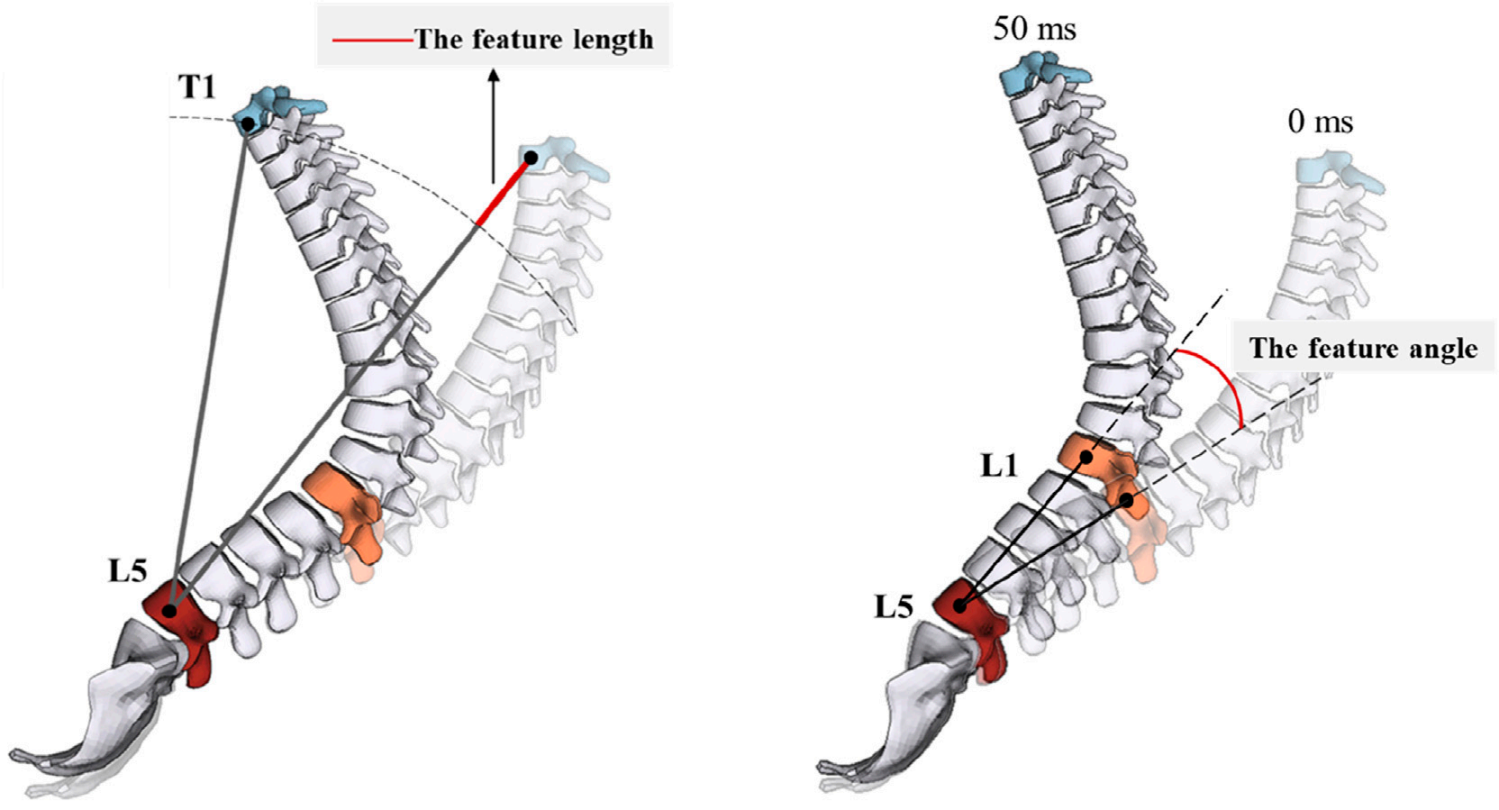
Definitions of the feature length and the feature angle.

For reclined occupant under frontal collision, since the overall deformation of the spine is highly correlated with the lumbar injury risk during the crash, this study proposes two geometric feature parameters, the feature length and the feature angle, to establish a link between the spinal kinematic response and the lumbar injury risk (Figure 10). The feature length is defined as the maximum variation in the distance between the T1 and L5 vertebrae in the XZ plane. This parameter primarily reflects the degree of overall axial deformation of the thoracolumbar spine and is determined by both structural compression and flexion. The feature angle is defined as the change in the angle between the line connecting the lumbar vertebrae L1 and L5 from 0 ms to 50 ms (optional). This parameter mainly reflects the degree of lumbar repositioning during the early stage of the collision.

Values of the feature length and feature angle for each case under 50 km/h are shown in Table 4. The feature length shows a strong correlation with the lumbar injury risk (Pearson’s correlation coefficient = 0.99). Furthermore, the feature angle effectively characterizes the performance of the assisted repositioning device, indicating the speed of the lumbar repositioning. The feature angle also correlates well with lumbar injury risk (Pearson’s correlation coefficient = 0.87). Thus, the feature parameters can well demonstrate the effectiveness of the lumbar protection strategy, which uses assisted repositioning to control the overall kinematics of the thoracolumbar spine within a safe mode.

**TABLE 4:** Geometric features and lumbar injury risks of each case.

Case	Feature length (mm)	Feature angle (°)	Lumbar injury risk
Normal Restraint	74.2	−1.9	66.9%
Inertial Repositioning	53.6	3.3	54.3%
Assisted Repositioning	14.9	15.4	32.4%
Coupled Restraint	47.8	13.4	51.8%

Although the highest lumbar injury risk was located at L1 in all the above cases, further investigation of lumbar compression forces and flexion moments revealed that the maximum values occurred at L5, except the normal restraint condition. Despite these high forces and moments, the injury risk at L5 remained the lowest among the lumbar vertebrae, primarily because the L5 vertebral body has a 50% greater cross-sectional area compared to that of L1, indicating a higher load-bearing capacity as reflected in the lumbar injury risk function. These results suggest that the protection concepts of inertial return and assisted repositioning also involve the idea of load transfer, where the collision load is shifted from the relatively fragile parts of the spine to the stronger parts, thereby reducing the overall risk of injury.

Spine protection is a new requirement for reclined occupant under frontal impact. Meanwhile, crash protection of reclined occupant also demands reconfiguration of seatbelt and airbag settings, especially when assisted repositioning is taken as main protection strategy. The repositioning must be started before or as soon as a collision begins and completed during the first stage of the occupant ride down process. Since seatbelt and frontal airbag are designed for upright posture, their restraining functions must be reconfigured in order to coordinate with the execution of occupant repositioning.

The three-point seatbelt system plays an important role in crash protection for upright occupants. However, as observed from the kinematic response, the presence of the shoulder-belt significantly impedes the occupant’s upper body from repositioning to the upright position, forcing the spine into a more dangerous deformation with increased flexion and compression. The simulations conducted in this study have clearly shown this negative effect, which also suggested that, at least during the first 50 ms of the crash response (optimal time depends on crash severity and other crash conditions), the restraint of the shoulder-belt should be minimized or even eliminated, allowing the upper body of the reclined occupant to return to an upright position as soon as possible, thereby reducing the lumbar injury risk. In contrast, the restraint of the lap-belt plays a major role in the early stages of collision by restraining the forward excursion of occupant’s hip, which is essential for both anti-submarining and repositioning. Once the upper body is close to an appropriate upright position, the shoulder-belt becomes essential to control occupant ride-down and provides protection for critical areas such as the head and the chest. Moreover, the lap-belt no longer needs to maintain its initial restraint strength, as the risk of submarining is significantly reduced. Consequently, lap-belt restraint strength can be decreased for the iliac protection.

Overall, to achieve comprehensive crash protection for reclined occupants, the assisted repositioning process must include a coordinated function of both the shoulder-belt and the lap-belt, as incorporated in the whole-body solutions presented in this study. The active solution has proven effective in providing frontal crash protection for a 50° reclined occupant under a 50 km/h - 30 g collision. With further optimization of the restraint system, we believe that the compromised injury outcomes (e.g., HIC, BrIC, CSDM, thorax AIS 3+ risk) of both the active and passive solutions could be improved. For instance, adding a frontal airbag could help reduce head rotation, implementing a seat-mounted seatbelt could allow for better optimization of the shoulder-belt restraint load, and applying variable seatbelt loads at different stages could improve ride-down efficiency during the repositioning process.

During the assisted repositioning process, we also considered the potential risk of injury from the repositioning device pushing the occupant’s back at high speed. Considering that the lumbar support/airbag force acts primarily in the X direction, we further analyzed the X-direction tangential force at the vertebral cross-section near the lumbar support position. The results showed no significant difference in the peak tangential force compared to the normal restraint condition. Therefore, it can be assumed that the impact of the repositioning device in this study does not pose an additional risk, although this requires further evaluation.

In production seats, the assisted repositioning device can be integrated into the seat backrest. For instance, the active lumbar support can be implemented using an electric motor to drive the existing lumbar support component, providing the necessary push for repositioning. The airbag-based solution offers an even simpler implementation, requiring only appropriately sized airbags installed at the corresponding location within the seat back. Additionally, multiple airbags can be arranged along the backrest in the lumbar region to accommodate occupants of varying body sizes. Future research can explore intelligent and adaptive application of the assisted repositioning device based on occupant body size (e.g., fifth, 95th percentiles), posture, and crash conditions.

## 5 Conclusion

In this study, a lumbar protection strategy termed “assisted repositioning” was proposed for reclined occupants in frontal collisions, building on the inertial repositioning method presented by Ji et al. (2017). For occupants in reclined angle of 50°, the effectiveness of the assisted repositioning has been demonstrated by comparing lumbar injury risks in multiple restraint settings. The assisted repositioning device can effectively reduce the lumbar injury risk under crash severities from 30 to 70 km/h, with the best effect around 50 km/h. To achieve whole-body crash protection, two virtual solutions were presented, and simulation results indicated that, with appropriate coordination between the assisted repositioning and the seatbelt system, the active solution could provide effective frontal crash protection for a 50° reclined occupant in a 50 km/h crash. With further optimization of the restraint system, we believe that whole-body safety for reclined occupants could be enhanced, potentially achieving a well-developed passive solution.

## Data Availability

The raw data supporting the conclusions of this article will be made available by the authors, without undue reservation.

## References

[B1] Baudrit P. Uriot J. Richard O. Debray M. (2022). Investigation of potential injury patterns and occupant kinematics in frontal impact with PMHS in reclined postures. Stapp Car Crash J. 66, 1–30. 10.4271/2022-22-0001 37733820

[B2] Baumann K. Schoneburg R. Justen R. (2001). The vision of a comprehensive safety concept. SAE. 10.1016/j.jbiomech.2022.111051

[B3] Boyle K. J. Reed M. P. Zaseck L. W. Hu J. (2019). “A human modelling study on occupant kinematics in highly reclined seats during frontal crashes,” in Proceedings of IRCOBI.

[B4] Dissanaike S. Kaufman R. Mack C. D. Mock C. Bulger E. (2008). The effect of reclined seats on mortality in motor vehicle collisions. J. Trauma Inj. Infect. and Crit. Care 64, 614–619. 10.1097/ta.0b013e318164d071 18332800

[B5] Forman J. Kulkarni S. Perez‐Rapela D. Mukherjee S. Panzer M. Hallman J. (2022). “A method for thoracic injury risk function development for human body models,” in Proceedings of IRCOBI.

[B6] Gepner B. D. Draper D. Mroz K. Richardson R. Ostling M. Pipkorn B. (2019). Comparison of human body models in frontal crashes with reclined seatback. Proceedings of *IRCOBI* .

[B7] Ji P. J. Huang Y. Zhou Q. (2017). Mechanisms of using knee bolster to control kinematical motion of occupant in reclined posture for lowering injury risk. Int. J. Crashworthiness 22, 415–424. 10.1080/13588265.2016.1275430

[B8] Mueller A. S. Cicchino J. B. Zuby D. S. (2020). What humanlike errors do autonomous vehicles need to avoid to maximize safety? J. Saf. Res. 75, 310–318. 10.1016/j.jsr.2020.10.005 33334489

[B9] Östh J. Bohman K. Jakobsson L. (2020). “Evaluation of kinematics and restraint interaction when repositioning a driver from a reclined to an upright position prior to frontal impact using active human body model simulations,” in Proceedings of IRCOBI.

[B10] Östling M. Lundgren C. Lubbe N. Huf A. Wernicke P. Pipkorn B. (2021). “The influence of a seat track load limiter on lumbar spine compression forces in relaxed, reclined, and upright seating positions: a sled test study using THOR-50m,” in Proceedings of IRCOBI.

[B11] Östling M. Lundgren C. Lubbe N. Pipkorn B. (2022). Reducing lumbar spine vertebra fracture risk with an adaptive seat track load limiter. Front. Future Transp. 3, 890117. 10.3389/ffutr.2022.890117

[B12] Östling M. Sunnevång C. Svensson C. Kock H. O. (2017). “Potential future seating positions and the impact on injury risks in a Learning Intelligent Vehicle (LIV) – how to avoid submarining in a reclined seating position in a frontal crash,” in Fahrzeugsicherheit, 261–276.

[B13] Pilet C. Vernet C. Martin J.-L. (2021). Estimated crash avoidance with the hypothetical introduction of automated vehicles: a simulation based on experts’ assessment from French in-depth data. Eur. Transp. Res. Rev. 13, 65. 10.1186/s12544-021-00521-2

[B14] Rawska K. Gepner B. Kulkarni S. Chastain K. Zhu J. J. Richardson R. (2019). Submarining sensitivity across varied anthropometry in an autonomous driving system environment. Traffic Inj. Prev. 20, S123–S127. 10.1080/15389588.2019.1655734 31539280

[B15] Richardson R. Donlon J.-P. Jayathirtha M. Forman J. L. Shaw G. Gepner B. (2020a). Kinematic and injury response of reclined PMHS in frontal impacts. Stapp Car Crash J. 64, 83–153. 10.4271/2020-22-0004 33636004

[B16] Richardson R. Jayathirtha M. Chastain K. Donlon J. P. Forman J. Gepner B. D. (2020b). Thoracolumbar spine kinematics and injuries in frontal impacts with reclined occupants. Traffic Inj. Prev. 21, S66–S71. 10.1080/15389588.2020.1837365 33206553

[B17] Richardson R. Jayathirtha M. Donlon J. P. Forman J. L. Gepner B. Ostling M. (2020c). “Pelvis kinematics and injuries of reclined occupants in frontal impacts,” in Proceedings of IRCOBI. 10.1080/15389588.2020.1837365 33206553

[B18] Shen W. Zhou Q. Tang J. (2023). “Pedestrian knee kinematics and injuries upon vehicle collision considering realistic pre-impact avoidance maneuvers,” in Proceedings of IRCOBI.

[B19] Sun Z. Lin M. Chen W. Dai B. Ying P. Zhou Q. (2023). A case study of unavoidable accidents of autonomous vehicles. Traffic Inj. Prev. 25, 8–13. 10.1080/15389588.2023.2255333 37722829

[B20] Takhounts E. Craig M. Moorhouse K. McFadden J. Hasija V. (2013). Development of brain injury Criteria (BrIC). Stapp car crash J. 57, 243–266. 10.4271/2013-22-0010 24435734

[B21] Tang J. Zhou Q. Shen W. Chen W. Tan P. (2023). Can we reposition finite element human body model like dummies? Front. Bioeng. Biotechnol. 11, 1176818. 10.3389/fbioe.2023.1176818 37265993 PMC10229860

[B22] Tushak S. K. Donlona J. P. Gepnera B. D. Chebbia A. Pipkornb B. Hallmanc J. J. (2022). Failure tolerance of the human lumbar spine in combined compression and flexion loading. J. Biomechanics 135, 111051. 10.1016/j.jbiomech.2022.111051 35325753

[B23] Uriot J. Masuda M. Trosseille X. Petit P. Compigne S. Richard O. (2015). Reference PMHS sled tests to assess submarining. Stapp Car Crash J. 59, 203–223. 10.4271/2015-22-0008 26660745

[B24] Woolley R. L. (2008). Crash pulse scaling applied to accident reconstruction. SAE Int. 10.4271/2008-01-0183

